# CKD-associated pruritus in haemodialysis: a road map for diagnosis and treatment

**DOI:** 10.1093/ckj/sfaf096

**Published:** 2025-04-24

**Authors:** Joerg Latus, Antoine Lanot, Sonja Ständer, Emilio Sanchez-Alvarez, Filippo Aucella, Gil Yosipovitch

**Affiliations:** Department of Nephrology and Internal Medicine, Robert-Bosch-Hospital, Stuttgart, Germany; Normandie Université, UNICAEN, CHU de Caen Normandie, Néphrologie, Caen, France; Department of Dermatology and Center for Chronic Pruritus (KCP), University Hospital Münster, Münster, Germany; Department of Nephrology, Hospital Universitario Central de Asturias, Oviedo, Spain; Department of Nephrology and Dialysis, Scientific Institut for Research and Health Care, Fondazione Casa Sollievo della Sofferenza, San Giovanni Rotondo, Italy; University of Miami Miller School of Medicine, Dr Phillip Frost Department of Dermatology and Cutaneous Surgery and Miami Itch Center, Miami, FL, USA

**Keywords:** chronic kidney disease, CKD-aP, diagnosis, haemodialysis, pruritus

## Abstract

Of the wide range of symptoms affecting patients with chronic kidney disease (CKD) on haemodialysis, CKD-associated pruritus is one of the most common and burdensome, occurring at moderate-to-severe intensity in 31%–40% of patients, significantly impacting multiple aspects of quality of life, and associated with increased healthcare utilization. Despite the distressing nature of this symptom, clinicians frequently underestimate its prevalence and it is under-reported by patients who may be unaware of the availability of effective treatment options. The identification and management of CKD-associated pruritus should form an essential aspect of patient-centred care; however, patients with CKD may have multiple causes of chronic itch including those of dermatological, systemic, neuropathic and psychogenic origin, and CKD-associated pruritus must be distinguished from these. Together with its highly variable presentation in patients on haemodialysis, the range of potential causes of itch makes differential diagnosis of CKD-associated pruritus challenging. The presence of bilaterally symmetrical and non-dermatomally distributed itching, commonly affecting the back, limbs, chest and head is characteristic of CKD-associated pruritus, although approximately 50% of patients report generalized pruritus. Secondary skin lesions (including excoriation, crusts, impetigo, lichenifications and prurigo also seen in dermatological conditions) may or may not be observed, and xerosis (dry skin) that may exacerbate itching is common. Here, we provide a pragmatic approach to the identification and differential diagnosis of chronic itching in CKD-associated pruritus with the aim of supporting the effective management of this highly distressing symptom in clinical practice.

## INTRODUCTION

Chronic kidney disease (CKD) is a global public health concern, significantly impacting morbidity and mortality, the burden of which increase as the disease progresses [1, 2]. Globally, approximately 3.9 million people were estimated to be living on kidney replacement therapy in 2017 [2], the majority of whom (∼69%) received haemodialysis [3]. These patients may experience a range of symptoms affecting quality of life [4, 5] with CKD-associated pruritus of moderate-to-severe intensity reported in up to 40% of patients and representing a particularly debilitating condition [6–8]. This distressing symptom may also be observed in patients on peritoneal dialysis [6] and in non-dialysis CKD [6, 9]. Despite the recognized impact of CKD-associated pruritus on quality of life [8], patients may under-report its presence and clinicians may significantly underestimate its prevalence, leading to undertreatment [10].

In line with recent calls to broaden the focus from haemodialysis efficiency to living well with kidney disease [11], there is a need to effectively identify and manage symptoms such as CKD-associated pruritus to improve patient experience and quality of life. However, the situation is complicated by the range of potential underlying causes of pruritus [12–15] that must first be excluded to ensure appropriate treatment can be provided. Previous publications have outlined a treatment algorithm for the management of CKD-associated pruritus [16–19]. Here, we present a pragmatic approach that extends and complements this algorithm to help nephrologists and the wider dialysis team identify and differentially diagnose itch in patients on haemodialysis, ultimately supporting its effective management.

## SYMPTOM BURDEN IN HAEMODIALYSIS

Patients undergoing haemodialysis may experience a broad range of physical and psychological symptoms among which itch is one of the most frequently reported [20]. Pruritus was one of the five most common symptoms identified in two studies of haemodialysis patients alongside pain, fatigue, dry skin, sleep problems (sleepiness/trouble staying asleep), difficulty concentrating and muscle cramps [4, 5]. Itching is also widely reported as among the most bothersome symptoms [5, 21, 22]. In the final analysis of the real-world retrospective, cross-sectional, multicentre CENSUS-EU study, overall prevalence of CKD-associated pruritus (of any severity) in patients undergoing haemodialysis (*N* = 2963) was 53.5%, and 31.2% of all patients experienced pruritus of moderate or severe intensity [23]. Similar results have been reported in the prospective international Dialysis Outcomes and Practice Patterns Study (DOPPS; Phases 4 to 6; 2009–18) in which 37% of 23 264 haemodialysis patients were at least moderately bothered by this symptom [8].

Not surprisingly considering the degree of bother, the presence of CKD-associated pruritus markedly impairs the quality of life of patients undergoing haemodialysis [10]. Studies have reported its association with mood changes and increasing rates of depression [6, 8, 9, 23–25], impairment of sexual activity [6], impact on social relations [10, 24] and poor sleep quality [6, 9, 23], with a greater impact on quality of life as the intensity of itching increases [23, 26]. CKD-associated pruritus also has an impact on healthcare utilization, with increasing pruritus severity associated with increased healthcare costs including increased use of medications such as antibiotics and antidepressants [27, 28] and increased hospitalization rates [8, 25]. An increased mortality risk has also been suggested [8, 25, 29]. As illustrated by a longitudinal study (*N* = 7976 haemodialysis patients in DOPPS), CKD-associated pruritus is a chronic condition, with 61% of patients at least moderately bothered by itching at baseline remaining at least moderately bothered when assessed after 1 year [25]. Only 10%–15% of these patients were receiving treatments supported by efficacy data from clinical trials of CKD-associated pruritus (gabapentin, pregabalin or nalfurafine) [25]. Similar chronicity has been observed in other studies, with one reporting continued negative effects on quality of life over 2 years of follow-up [30]. Data such as these highlight the importance of effective identification and management of this potentially debilitating symptom in patients on haemodialysis.

## CLINICAL PRESENTATION OF PRURITUS IN PATIENTS ON HAEMODIALYSIS AND DIFFERENTIAL DIAGNOSES

### Screening for pruritus

**Figure 1:**
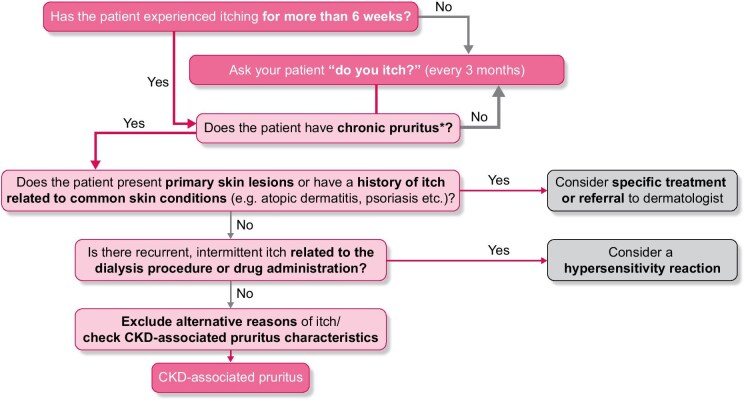
The differential diagnosis of CKD-associated pruritus. *Defined as an unpleasant sensation of the skin leading to the desire to scratch, with symptoms present for more than 6 weeks.

Diagnosing and ultimately alleviating the burden of CKD-associated pruritus requires the proactive identification of patients who suffer from it (Fig. 1). Proactive questioning is essential as clinicians under-estimate itch prevalence [10, 31], and often do not adequately treat the condition [32]. In a recent study of patients with moderate-to-very-severe pruritus, healthcare staff were unaware of the condition in 38% (114/303) of cases [26]. CKD-associated pruritus can also be under-reported by patients [31]. Some potential reasons for this include a lack of understanding of the relationship with CKD, patient acceptance of the symptom as something they have to live with, limited patient awareness of treatment options and concerns that health professionals may not consider itch to be a problem [10, 33]. Regular screening (every 3 months) therefore ensures that patients who have newly developed CKD-associated pruritus can be recognized and supported [17–19]. The chronicity of the condition should then be determined: chronic pruritus can be defined as an unpleasant sensation of the skin leading to the desire to scratch, with symptoms present for more than 6 weeks [14, 34].

### Clinical presentation of CKD-associated pruritus

**Figure 2:**
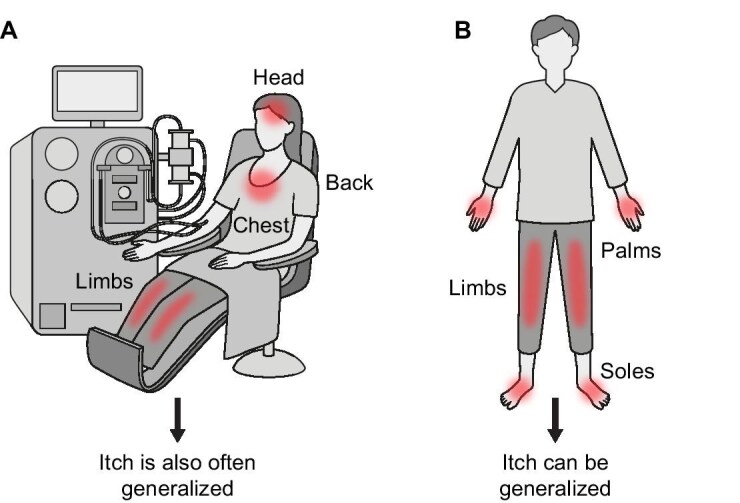
(**A**) Typical presentation of pruritus associated with CKD. (**B**) Typical presentation of pruritus in hepatobiliary disease.

As chronic pruritus may be associated with a number of different underlying causes [12, 15], alternative explanations for this symptom must be excluded before a diagnosis of CKD-associated pruritus can be made [14, 15]. This differential diagnosis can be challenging because of the variable presentation among patients with CKD-associated pruritus and the presence of non-specific symptoms (Table 1). Although itch may start prior to initiation of haemodialysis [6, 9], it typically begins in the months after dialysis [29] and persists over time [25, 30]. Pruritus can be intermittent or continual, and its intensity can vary over time: in DOPPS (*N* = 35 452 haemodialysis patients), among patients bothered by itchy skin, nearly 50% reported being most bothered by itching either at all times throughout the day or not at any specific time, while approximately one-third found itching most bothersome at night [10]. Itching can occur at any time in relation to dialysis, with patients reporting aggravation, reduction or no effect on itching intensity during the process [35]. Itching is typically bilaterally symmetrical and non-dermatomally distributed (∼80% of patients [24]), commonly affecting the back, limbs, chest and head, although approximately 50% report generalized pruritus [6, 13, 26] (Fig. 2A). The spatial distribution can change over time [36] and more areas of the body can become involved as itch severity increases [6]. Skin lesions may not be noticeable [12, 19, 37], but where observed, excoriations are bilaterally symmetrical [24] and are generally only seen where the patient can reach to scratch so that scratch marks and nodules may form a butterfly shape on the back (sparing the area that the patient cannot reach [38]). However, as patients may use scratching aids to help relieve their itching, this pattern is not universally observed. This highly variable presentation underlies the challenges and importance of differential diagnosis.

**Table 1:** Clinical characteristics of CKD-associated pruritus.


Timing of itch onset	• Typically occurs after onset of dialysis [29] but may occur pre-dialysis [6, 9]
Symptom intensity and duration	• From mild to very severe [8]
	• Intermittent or continual (daily) occurring at any time of day and often worse at night [6, 10]
	• Persists over time [25, 30]
Impact of dialysis	• Highly variable between patients: no effect, aggravation or reduction of itching while on dialysis reported [35]
Location of itch	• Characteristic bilaterally symmetrical, non-dermatomal distribution [6, 24]
	• Back, limbs, chest and head typically affected but may be generalized [6, 13]
	• May be localized to the shunt arm [48, 89]
	• More areas affected as itch persists and intensifies [6]
Presence and patterns of skin lesions	• Usually starts on non-lesional skin and may lack prominent skin lesions during the course [12, 19, 37]
	• Dry skin (xerosis) is frequent and may aggravate itch [35, 36]
	• Secondary lesions due to scratching (e.g. excoriation, crusts, impetigo, lichenifications and prurigo) may be observed [36, 37, 40]
	• If present, skin lesions are typically bilaterally symmetrical [6, 24, 35] and may present as a butterfly pattern on the back [38] (only where patients can reach)

### Differential diagnosis of the cause of pruritus

Potential underlying causes of chronic itch include those of dermatological, systemic, neuropathic and psychogenic origin [12, 15, 34] (Table 2). A detailed review of the patient's entire medical history, along with clinical examination, is key to determining the aetiology of CKD-associated pruritus [14].

**Table 2:** Differential diagnosis of the cause of pruritus in haemodialysis patients.

Category	Cause
Dermatological	Atopic dermatitis
	Psoriasis
	Chronic urticaria
	Contact dermatitis
	Dermatophytosis
	Scabies infestations
	Bullous pemphigoid
Dialysis or drug related	Type of dialysis membrane or sterilizing agent
	Adverse/hypersensitivity reaction to concomitant medication
Systemic	CKD
	Hepatobiliary diseases, e.g. primary biliary cirrhosis
	Diabetes mellitus
	Hyperthyroid disease
	HIV infection
	Hepatitis C infection
	Haematological diseases, e.g. polycythemia vera
	Hodgkin lymphoma
	Non-Hodgkin lymphoma
	Hypercalcaemic states
Neuropathic	Small-fibre neuropathies
	Post-herpetic neuralgia
	Brachioradial pruritus
	Notalgia paraesthetica
	Scalp itch
Psychogenic	Obsessive compulsive disorder
	Substance abuse
	Delusions of parasitosis

The initial approach to differential diagnosis should be to check whether the patient has a history of itch related to common dermatological conditions and careful evaluation of the skin to distinguish primary skin lesions (those originating from the causal disease) from secondary skin lesions (reactive lesions induced by manipulations such as rubbing or scratching of the skin due to chronic itching) [14, 34, 39]. Dermatological causes of itching such as atopic dermatitis, psoriasis, chronic urticaria, dermatophytosis, scabies infestations, bullous pemphigoid, etc. (Table 2) should thus be ruled out [12]. While skin appears to be normal in a proportion of patients with CKD-associated pruritus [37], it is important to note that secondary lesions similar to those seen in dermatological conditions (e.g. excoriation, crusts, impetigo, lichenifications and prurigo) are commonly observed (Table 1) so do not necessarily represent criteria for exclusion [12, 37, 40]. Also of note is that xerosis (dry skin) is common in end-stage kidney disease, being seen in 50%–90% of patients, and can coexist and potentially exacerbate CKD-associated pruritus [34, 36, 40, 41].

As a next step, recurrent intermittent itch related to the dialysis procedure itself or to drug administration should be excluded. The use of some dialyser membranes has been associated with hypersensitivity reactions and switching to a more biocompatible membrane has therefore been suggested [42]. However, reactions have still been reported following the use of the high-flux polyacrylonitrile membrane AN69, as well as polysulfone and polyamide membranes. Reactions are typically seen shortly after starting dialysis and may be mild to severe [43]. The permeability and adsorption properties of the membrane and its ability to remove uraemic toxins may also be relevant for the presence of itch: polymethylmethacrylate filters that are hypothesized to adsorb ionic substrates into their polymer composition have been associated with beneficial effects on pruritus [44, 45]. In addition, reactions associated with sterilizing agents such as ethylene oxide and formaldehyde—typically occurring within the first 30 min of dialysis—may be seen in some patients [43].

Patients on haemodialysis have a high comedication burden and pruritus may result from an adverse drug reaction or hypersensitivity reaction [40, 43, 46]. Recently started, as well as long-standing, pharmacological therapy should be considered [47]. A recent prospective observational study noted that almost 50% of patients with moderate-to-severe CKD-associated pruritus were taking treatments that may be associated with itching, of which the use of statins was most common [26]. Hypersensitivity reactions to agents including iron, erythropoietin, heparin, topical antibiotics and anaesthetics have been reported [43]. Of note is that drugs commonly used in CKD such as angiotensin-converting enzyme inhibitors, clonidine, calcium antagonists, beta-blockers, diuretics and allopurinol may induce itch by activating mu-opioid receptors [48]. To avoid any negative effects of unnecessarily stopping a drug, it is important to carefully assess the temporal relationship between administration of the suspected drug and itching before undertaking a trial withdrawal period to assess its contribution.

Following exclusion of itch related to the dialysis procedure itself or an adverse/hypersensitivity reaction, other common comorbidities in CKD which can induce pruritus (in the absence of a primary skin lesion) must be ruled out [12, 15]. As such, it is crucial to perform a physical examination to evaluate symptoms and signs suggestive of systemic disease. Liver disease and diabetes are frequently observed in CKD. Itching associated with hepatobiliary diseases is often localized at the distal extremities such as the palms of the hands and soles of the feet [13], which are not typically affected in CKD-associated pruritus [39] (Table 1; Fig. 2). These patients may also present with typical signs of hepatobiliary disease such as jaundice or purpura although itching may precede these [12]. In patients with diabetes mellitus, xerosis and diabetic polyneuropathy are associated with itch, which can be localized and generalized [49, 50]. Localized itching occurs mainly on the lower extremities, back and scalp [50]. A number of other systemic conditions such as hyperthyroid disease [13], primary biliary cirrhosis [12], HIV infection [12], hepatitis C infection [12], myeloproliferative neoplasms including polycythemia vera [12, 13], Hodgkin lymphoma, non-Hodgkin lymphoma [13] and cutaneous T-cell lymphoma [13] should be considered as potential causes of pruritus. In polycythemia vera, contact with water can cause severe itching with no skin manifestations (aquagenic pruritus), and Hodgkin lymphoma may be associated with itching at night accompanied by weight loss, fevers and night sweats [51]. Of note is that itch has been reported as a presenting symptom in the late stages of cutaneous T-cell lymphoma mycosis fungoides, a condition which is potentially life threatening [52]. Various laboratory tests and investigations are available to aid nephrologists and the wider dialysis team in identifying systemic diseases in certain cases. These include complete blood count; assessment of thyroid-stimulating hormone, blood urea nitrogen, creatinine, glucose, ferritin, C-reactive protein, calcium and parathormone levels; liver function tests; evaluation of autoimmune disease markers; virus and parasite serology; skin scraping for scabies; and a chest X-ray [14]. However, it should be noted that these complementary tests and investigations aid in the differential diagnosis of CKD-associated pruritus by excluding systemic diagnoses rather than directly identifying the condition itself.

Lastly, potential neuropathic and psychogenic causes of itch (in the absence of primary skin lesions) should be considered. Neuropathic itch (defined as an itch initiated or caused by a primary lesion or dysfunction at any point along the afferent pathway of the nervous system [53, 54]) is associated with conditions such as postherpetic neuralgia (occurring at the site of prior infection), brachioradial pruritus (characterized by itching of the arms), notalgia paraesthetica (characterized by an itch of the upper back) and scalp itch [12, 39, 53, 54]. Neuropathic itch is often severe [53] and may coincide with pain or other symptoms such as sensory loss in a dermatomal distribution [39, 53, 54], thus making it distinct from the non-dermatomal pattern usually seen with CKD-associated pruritus [24]. Neuropathic itch is typically localized at the area of damage/dysfunction [39], but can be more generalized, particularly if associated with nerve fibre degeneration [53]. Psychogenic pruritus is characterized by an excessive impulse to scratch, gouge or pick at normal skin and is seen in conditions including obsessive-compulsive disorder, substance abuse or delusions of parasitosis [54]; these should be ruled out.

In patients with typical symmetrical pruritus and no indication of a primary lesion after excluding all other potential aetiologies, nephrologists can proceed with diagnosing CKD-associated pruritus and initiating treatment without consulting a dermatologist. However, in more challenging cases, referral to a dermatologist should be considered [48]. Such cases include patients presenting with dermatological causes of pruritus; cases where it is difficult to distinguish primary from secondary lesions; and patients presenting with nonspecific dermatological findings such as prurigo lesions, eczematous lesions or comorbid dermatoses, which may require further evaluation.

## TREATMENTS USED TO MANAGE CKD-ASSOCIATED PRURITUS (OFF- AND ON-LABEL)

**Figure 3:**
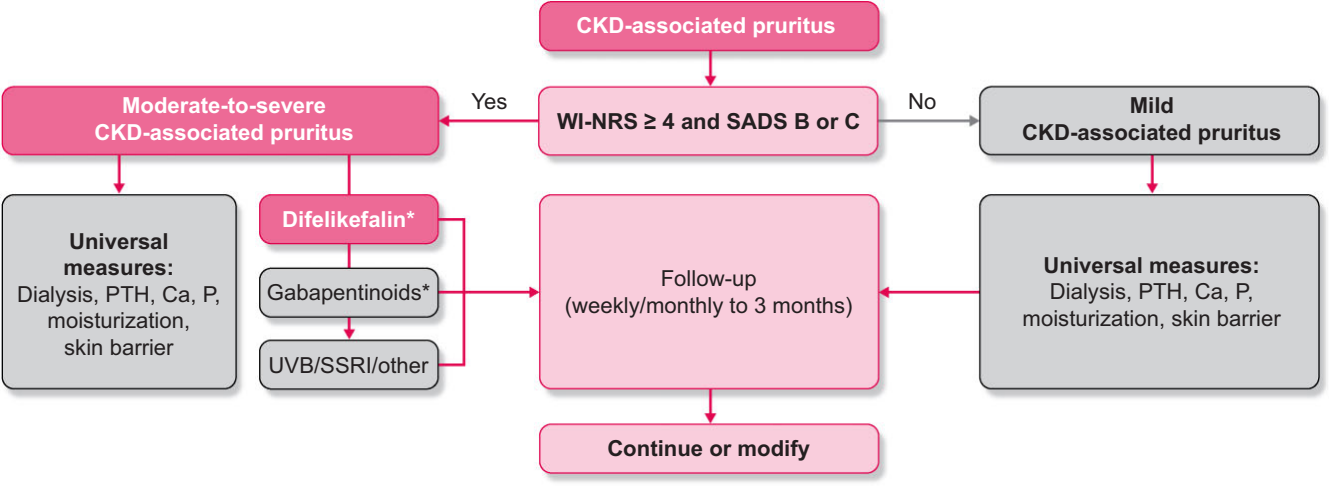
A treatment algorithm for CKD-associated pruritus (adapted from Agarwal *et al*. *Clin Kidney J*, 2023 [17]). *Treatment selection dependent on the availability of difelikefalin. Ca, calcium; P, phosphorus; SSRI, selective serotonin reuptake inhibitor.

Following exclusion of other causes and a diagnosis of CKD-associated pruritus, simple validated tools should be used to assess both itch intensity and its impact on the patient's quality of life: the combination of these assessments is important to determine appropriate management (Fig. 3). A number of patient-reported outcome tools are available and have recently been reviewed [19, 48]. Of these, the use of the Worst Itch Intensity Numeric Rating Scale (WI-NRS) together with the Self-Assessed Disease Severity (SADS) tool has been suggested to assess CKD-associated pruritus in several publications as they are simple and quick to use and therefore well suited to routine clinical practice [16–18, 48]. Briefly, the WI-NRS is a unidimensional quantitative tool for measuring pruritus intensity that has been validated in clinical trials [24, 55]. Patients are asked to score the intensity of their worst itching over the previous 24 h on a scale from 0 (no itching) to 10 (worst possible itching), with WI-NRS scores generally categorized as mild (<4), moderate (≥4–6) or severe/very severe (≥7) itching [56]. The SADS is a multidimensional qualitative tool which measures the impact of pruritus on quality of life, asking a patient to categorize themselves into one of three different types (A–C) reflecting increasing signs and symptoms associated with the condition [24]. Used together, the WI-NRS and the SADS allow a patient's itching to be classed as mild (WI-NRS score <4; SADS patient type A) or moderate to severe [WI-NRS score ≥4–6 (moderate) or ≥7 (severe); SADS patient type B or C] [17, 18, 48].

For those patients categorized as having mild CKD-associated pruritus, universal management measures should be optimized [16–18, 48]. As noted previously, dry skin is common in patients on haemodialysis [41] and may contribute to the intensity of itching. Skin moisturization with emollients or topical creams may provide some degree of relief [35, 57, 58]. Optimal dialysis should be ensured: increasing the dialysis dose to more effectively remove uraemic toxins may improve itching in a proportion of patients [59–61], although the current benefits of this when target Kt/V is now routinely achieved in clinical practice is unclear [26]. A change in dialyser membrane [43, 44] or a switch from conventional to high-flux dialysers have been reported as beneficial and can be considered [62, 63]. Due to limited data suggesting a lower prevalence of CKD-associated pruritus in patients on peritoneal versus haemodialysis, a change of dialysis modality has been suggested as a possible management strategy [64]; however, evidence from recent reports is conflicting [57]. For example, in a Dutch longitudinal study of haemodialysis (*N* = 1256) and peritoneal dialysis (*N* = 670) patients, CKD-associated pruritus of at least mild severity was observed in 70% and of at least moderate severity in 29% of patients, regardless of modality [65].

The association between markers of mineral metabolism [calcium, phosphorus and parathyroid hormone levels (PTH)] and CKD-associated pruritus is unclear with conflicting reports in the literature [19] and no or limited relationship between these parameters and pruritus severity was found in recent analyses [8, 26]. However, given the uncertainty of their contribution and the importance of achievement of guideline targets for calcium, phosphorus and PTH in the management of CKD–metabolic bone disease [66], optimizing management of these parameters is a reasonable step [16–19].

For patients with moderate-to-severe CKD-associated pruritus (WI-NRS ≥4 or SADS B or C), the universal measures described above should also be optimized, although further treatment will generally be required to address this severely distressing condition [46]. Despite the impact on quality of life, the data supporting many of the treatments traditionally used are sparse and often conflicting [67], and most are prescribed off-label. An in-depth analysis of the strengths and limitations of these various treatments when used in CKD-associated pruritus are beyond the scope of this article and have been reviewed elsewhere [19, 57, 67, 68]; a summary of the utility of commonly used treatments is provided here.

Difelikefalin is a selective, peripherally restricted kappa opioid receptor agonist developed to address the imbalance in the endogenous opioid system (e.g. overexpression of mu opioid receptors and concomitant downregulation of kappa opioid receptors) implicated in the pathogenesis of pruritus [36, 57, 69–71]. Uniquely, difelikefalin has been approved for the treatment of moderate-to-severe CKD-associated pruritus in adult haemodialysis patients in a number of countries including the USA and Europe based on the results of two large randomized, placebo-controlled Phase 3 trials (*N* = 851) [69, 72, 73]. Intravenous administration of difelikefalin at the end of haemodialysis three times a week for 12 weeks led to a significantly greater proportion of patients achieving a clinically significant change from baseline (≥3-point reduction) in WI-NRS score versus placebo (*P *< 0.001 at 12 weeks) [72]. Associated clinically relevant improvements in itch-related quality of life measures including sleep quality have also been reported [72, 74–76], and these results have now been supported by real-world evidence [77]. Difelikefalin is generally well tolerated with mild-to-moderate adverse events that typically resolve over time and no abuse potential or signs of physical dependence have been reported [71, 78, 79]. It should therefore be considered as a first-line treatment option for moderate-to-severe CKD-associated pruritus in countries where it is available [80]. Nalfurafine is another selective kappa opioid receptor agonist that has shown benefits in randomized, placebo-controlled studies of CKD-associated pruritus [67, 81, 82]; however, it is only licensed for use in Japan, South Korea and China [83, 84].

The use of gabapentinoids in CKD-associated pruritus is common and a systematic review of five small studies (total *N* = 297) concluded that gabapentin or pregabalin were effective in reducing itch compared with placebo [67], although these agents are not licensed for use in this indication. In addition, gabapentinoids present an increased risk of a number of adverse events including dizziness and somnolence which are commonly seen and, in rare cases, suicidal ideation/behaviour and anaphylaxis [85]. In haemodialysis patients, gabapentin and pregabalin have been associated with a risk of altered mental status, falls and fracture [86], which are of particular concern in this population who are often >60 years of age and already frail [87]. Gabapentinoids should therefore be used with caution for CKD-associated pruritus: titration from low starting doses, careful monitoring for adverse events and patient education of the potential side effects are required [19, 66]. Where difelikefalin treatment is available, it may be prudent to reserve gabapentinoid use for cases of insufficient response or intolerance to difelikefalin [16–18].

Although antihistamines are widely prescribed in CKD-associated pruritus [10], the itch in this population is mediated by nonhistaminergic nerve fibres [13, 64] and there is no robust evidence to support their use with clinical trials failing to demonstrate efficacy [67, 68]. Instead, the sedative effect of these agents may contribute to the perceived benefits [19]. Antihistamines are therefore not recommended as a treatment for CKD-associated pruritus, unless the sedative effects are required to aid sleep at night [39, 68]. Other off-label agents that have shown some reduction in itching in clinical trials of CKD-associated pruritus include the leukotriene receptor antagonist montelukast [67], the anti-depressant agent sertraline [68] and the serotonergic antagonist mirtazapine [88]. However, the evidence for these agents is restricted to small trials and further studies are required to confirm their benefits. Non-pharmacological approaches to the management of CKD-associated pruritus include ultraviolet B (UVB) phototherapy and acupuncture which have both been found to provide some benefit in small and/or uncontrolled studies [67, 68]. Prior to UVB treatment, the risk of skin cancer in this population, particularly if immunosuppressed, should be considered [68].

### The importance of regular symptom assessments

Even once the diagnosis of CKD-associated pruritus has been made and treatment for itch is ongoing, with the high symptom burden experienced by patients on dialysis, it is of critical importance that the renal care team regularly and proactively assess whether treatments for itching are providing relief and consider adaptations to management if required. Ongoing management will need to consider regional variations in the availability of different treatment options alongside an individualized approach focusing on individual patient characteristics and needs [19].

## CONCLUSIONS

For patients with CKD on haemodialysis, effective symptom management should be considered a priority of patient-centred care [11]. Of the wide range of symptoms affecting these patients, CKD-associated pruritus is particularly common and debilitating, significantly impacting the quality of day-to-day life [6, 8, 10]. Uniquely among other symptoms, it now has a specifically approved therapy for adults on haemodialysis [73]. However, its prevalence is underestimated by clinicians, it is under-reported by patients [10, 26] and, where identified, it may be ineffectively treated with off-label agents such as antihistamines [10, 26]. We hope that providing the clinical community with pragmatic algorithms to proactively identify and manage patients suffering from pruritus will increase the use of effective treatments and ultimately lead to improved patient quality of life and satisfaction with their care.

## Data Availability

No new data were generated or analysed in support of this research.
